# A novel biomarker of amnestic MCI based on dynamic cross-frequency coupling patterns during cognitive brain responses

**DOI:** 10.3389/fnins.2015.00350

**Published:** 2015-10-20

**Authors:** Stavros I. Dimitriadis, Nikolaos A. Laskaris, Malamati P. Bitzidou, Ioannis Tarnanas, Magda N. Tsolaki

**Affiliations:** ^1^Artificial Intelligence Information Analysis Lab, Department of Informatics, Aristotle University of ThessalonikiThessaloniki, Greece; ^2^Neuroinformatics Group, Department of Informatics, Aristotle University of ThessalonikiThessaloniki, Greece; ^3^Health-IS Lab, Chair of Information Management, ETH ZurichZurich, Switzerland; ^4^3rd Department of Neurology, Medical School, Aristotle University of ThessalonikiThessaloniki, Greece

**Keywords:** cognitive impairment, ERPs, phase-amplitude coupling, functional connectomics, dynamic coordination, dynome, connectomic biomarkers

## Abstract

The detection of mild cognitive impairment (MCI), the transitional stage between normal cognitive changes of aging and the cognitive decline caused by AD, is of paramount clinical importance, since MCI patients are at increased risk of progressing into AD. Electroencephalographic (EEG) alterations in the spectral content of brainwaves and connectivity at resting state have been associated with early-stage AD. Recently, cognitive event-related potentials (ERPs) have entered into the picture as an easy to perform screening test. Motivated by the recent findings about the role of cross-frequency coupling (CFC) in cognition, we introduce a relevant methodological approach for detecting MCI based on cognitive responses from a standard auditory oddball paradigm. By using the single trial signals recorded at Pz sensor and comparing the responses to target and non-target stimuli, we first demonstrate that increased CFC is associated with the cognitive task. Then, considering the dynamic character of CFC, we identify instances during which the coupling between particular pairs of brainwave frequencies carries sufficient information for discriminating between normal subjects and patients with MCI. In this way, we form a multiparametric signature of impaired cognition. The new composite biomarker was tested using data from a cohort that consists of 25 amnestic MCI patients and 15 age-matched controls. Standard machine-learning algorithms were employed so as to implement the binary classification task. Based on leave-one-out cross-validation, the measured classification rate was found reaching very high levels (95%). Our approach compares favorably with the traditional alternative of using the morphology of averaged ERP response to make the diagnosis and the usage of features from spectro-temporal analysis of single-trial responses. This further indicates that task-related CFC measurements can provide invaluable analytics in AD diagnosis and prognosis.

## Introduction

Alzheimer's disease (AD) is a neuro-degenerative disorder, characterized by loss of memory and declined cognitive and intellectual abilities, that severely affects not only patients' social life but even their daily living. Currently, the diagnosis of AD is performed via clinical neuropsychological tests with accuracies ranging from 85 to 93%. However, this widely-used procedure requires long sessions in hospitals and the involvement of experienced staff (Paajanen et al., 2014). For this reason, the definition of a reliable, low cost and, preferably, non-invasive biomarker for the early diagnosis of AD is an active research area. Toward this end electroencephalography (EEG) has been adopted as a potential screening method, since functional alterations due to AD most probably are reflected in the recorded cerebral activity of a patient (Ponomareva et al., 2013).

From the methodological side, the existing approaches fall in either of the two main streams in brain signal analysis: spectral and nonlinear dynamics (Dauwels et al., 2010, 2011). Regarding the first and most popular trend, earlier studies have demonstrated increased brain activity for δ (0.1–4 Hz) and θ (4–8 Hz) frequency bands and decreased activity for α (8–12 Hz) and β (12–30 Hz) frequency bands in AD patients (Cibils, 2002; Dauwels et al., 2010). In terms of brain connectivity, a reduced inter-hemispheric coherence for both α and β bands has been associated with AD (Dunkin et al., 1994; Locatelli et al., 1998). In addition to the alterations observed in the characteristics of brainwaves (spectral power and coherence), a correlation with the severity of disease has been demonstrated as well (Kowalski et al., 2001). In a more recent study, the quantification of cross-frequency amplitude-to-amplitude modulations during resting-state was introduced as a means of differentiating patients with mild AD symptoms from patients with moderate symptoms (Fraga et al., 2013).

Mild cognitive impairment (MCI) is considered as the transitional state between normal cognitive decline due to normal aging and the cognitive decay caused by AD. MCI subjects are in general at higher risk of suffering from dementia, where an extensive variation in annual conversion rates to AD was observed in many studies ranging from 10.2 to 33.6% (Espinosa et al., 2013). The neuropathology of MCI may exhibit the complex features of the early stages of AD, such as genomic alterations, plaque formation, changes in protein metabolism, synaptic dysfunction, and cellular injury (Stephan et al., 2012). There are increased research efforts to define a methodology for the reliable detection of MCI patients (Young et al., 2013). The timely identification of such patients provides the clinicians with the opportunity to organize therapeutic pharmaceutical treatment (Doody et al., 2014) or alternative interventions like serious gaming (Mosimann et al., 2004; Bahar-Fuchs et al., 2013; Tarnanas et al., 2014b; Muscio et al., 2015; Tarnanas et al., 2015a,b) and neurofeedback training (Fernández et al., 2008; Berman and Frederick, 2009; Becerra et al., 2012) at the very early stages of the AD, well before the neurogenerative processes have pushed the cognitive substrate to the “point of no return.” The early detection of MCI patients can enhance a positive response to therapy. The last decade different subtypes of MCI has been recognized, and a recommended diagnostic strategy is to technically dichotomize the patients into those of amnestic type (aMCI) and non-amnestic ones (Winblad et al., 2004). The aMCI patients form a cohort of particular clinical importance, due to the associated high conversion rate to AD (6 times higher risk than the age-matched controls) and the empirical observation of similar neuropathological findings with patients at early AD stage (Petersen et al., 1999).

The majority of MCI patients exhibit, at first, a cognitive decline in episodic memory. Apart from related neuropsychological clinical screening tests (e.g., MMSE), various neuroimaging techniques such as functional magnetic resonance imaging (fMRI), volumetric magnetic resonance imaging (vMRI), and positron emission tomography (PET) are also employed for the clinical diagnosis of MCI (Ewers et al., 2010; Patterson et al., 2011). On the contrary, EEG has not been widely incorporated into clinical practice as a diagnostic tool for detecting MCI and the rate of subsequent progression to AD. There is however a certain amount of published research work (Babiloni et al., 2010; Başar et al., 2013). This includes studies of functional connectivity as reviewed in Wen et al. (2015) and a few quantitative EEG (qEEG) studies reporting MCI-related alterations in the spectral characteristics of the recorded brain signal (Jelic et al., 2000; Moretti et al., 2012).

Recently, cognitive ERP studies have gained popularity for understanding and revealing dementive disorders (Frodl et al., 2002; Polich and Corey-Bloom, 2005; Jackson and Snyder, 2008; Papaliagkas et al., 2008, 2011; Lai et al., 2010; Missonnier et al., 2010; Laskaris et al., 2012), since they can target at specific mental faculties (the same that are scrutinized via neuropsychological screening) with the additional advantage of leading to directly quantifiable indices. Among the possible experimental designs (Olichney et al., 2002; Güntekin et al., 2013; Gozke et al., in press), the standard auditory oddball stimulus paradigm for Audiroty Event Related Potentials (AERPs) is the simplest to perform. After averaging the brain responses from the AERPs when targeting auditory tones, two of the main morphological components (known as N200 and P300 based on their polarity and latency) appear deteriorated in the case of MCI patients (Ritter et al., 1984; Golob et al., 2001). P300 is an endogenous brain response that occurs as a positive deflection, roughly, 300 ms after the onset of stimulus whenever the subject detects a meaningful task-relevant stimulus (Polich and Corey-Bloom, 2005). N200 is a faster component that reflects cognitive processes of stimulus evaluation, selective attention, and conscious discrimination (Patel and Azzam, 2005). Despite the well-recognized difficulty in identifying the neurophysiological origin of these deflections (Polich, 2007), the related latency and amplitude measurements are considered to suffice for assessing cognitive decline (Muscoso et al., 2006; Bennys et al., 2007; Golob et al., 2007; Caravaglios et al., 2008). More recently, the characteristics of event-related oscillations participating in the AERP response were brought into focus, with promising results regarding the clinical evaluation of MCI/AD (Yener et al., 2012; Yener and Basar, 2013).

Here, we delve into the event related oscillations and scrutinize further the underlying mechanisms so as to identify novel signatures of cognitive impairment. In particular we study the functional interactions between distinct brain rhythms, based on traces of oscillatory activity derived by filtering the single-trial responses within the standard frequency bands (δ, θ, α_1_, α_2_, β_1_, β_2_, γ_1_). The motivation came from the -recently established in neuroscience- concept that CFC is a key mechanism for the integration of distinct processes mediated by the distinct brain rhythms, and the rapidly accumulating experimental evidence about its role in cognition (Jensen and Colgin, 2007; Canolty and Knight, 2010; Palva and Palva, 2011; Buzsáki and Watson, 2012; Jirsa and Müller, 2013; Dimitriadis et al., 2015a). Four different types of CFC are usually mentioned (Jensen and Colgin, 2007), with each one considering a particular interaction mode (power to power, phase to phase, phase to frequency, and phase to power) between two distinct brain rhythms. Among these the fourth scenario, according to which the amplitude of a brain rhythm is modulated by the phase of a lower-frequency rhythm, is the one most often confirmed by experiments (Tort et al., 2008, 2009, 2010; Cohen et al., 2009a,b; Colgin et al., 2009; Axmacher et al., 2010a,b; Voytek et al., 2010).

For this work, we adopted a phase-to-amplitude (PAC) estimator and quantified the CFC between event-related oscillations recorded at the Pz electrode during an AERPs paradigm so as to test the hypothesis that the cognitive responses in aMCI patients are associated with deviations from a “normal” profile of interactions between brain rhythms. For this reason the CFC was measured in the AERPs responses of non-impaired (NI) elderly subjects as well. Since there had been no previous work on the particular topic, we followed different stages of analysis so as to verify that PAC estimates could lead to potential descriptor(s) of cognitive response dynamics and indicators of impairment. First, we examined the CFC in the case of normal subjects and demonstrated, by contrasting the responses to target and not-target stimuli that cognitive responses are associated with higher PAC levels. That stage of analysis revealed the transient and multifaceted nature of cross-frequency interactions that called for sophisticated analysis that could handle the dynamic nature of the examined phenomena. Next, we adopted the approach of evolving (i.e., time-varying) patterns of function interactions and formulated the search for a PAC-based biomarker as a pattern analytic task. Following a statistical learning scheme (that operated toward maximizing the discrimination between the aMCI patients and controls), we then selected the time instants and particular frequency-pairs that should be incorporated in building an effective biomarker. Finally, we employed a standard classifier so as to quantify the actual performance of the proposed PAC-based profile in aMCI detection. In addition, the overall learning scheme was repeated using morphological characteristics of the averaged AERP responses and spectro-temporal characteristics derived via single-trial analysis. The attempted comparison showed that the introduced approach not only provides new insights to the neuronal substrate of impaired cognition, but also outperforms the conventional data-analytics employed in ERP analysis.

The structure of the remaining paper is as follows. Section Materials and methods start by introducing the data and proceeds by introducing the PAC estimator as adopted for the purposes of this work. Results section includes the main results of our study, while some additional results have been appended as Supplementary Material. The final section is devoted to the discussion of the results and the future perspectives of this work.

## Materials and methods

### Subjects

Twenty-five amnestic MCI (aMCI) patients composed the MCI group (mean age ± std = 70 ± 5 years). The elderly (control) group consisted of a total of 15 healthy individuals with a similar range of ages to MCI group. All the participants provided written, informed consent. The subjects were selected over a pool of subjects that visit for regular interventions the Day Centre of Greek Association of Alzheimer Disease and Relative Disorders (GAADRD). The Ethics Committee of the Greek Association of Alzheimer Disease and Relative Disorders approved this study.

All subjects were assessed with a standardized neuropsychological test battery and aMCI was diagnosed using the following criteria: (1) memory complaint, (2) abnormal memory for age, (3) normal activities of daily living, (4) normal general cognitive function, and (5) not demented (Tarnanas et al., 2015b). Conspicuous brain abnormalities that could account for cognitive decline were excluded using structural magnetic resonance imaging (MRI) data. The baseline neuropsychological evaluation covered the following cognitive domains: episodic and working memory, attention/psychomotor processing speed, executive function, language, and visual-constructive abilities. Impairment was determined if at least one score per domain was 1.5 SD below group means compared to test-specific normative data (Petersen and Morris, 2005). The overall evaluation typically included one or more composite or global measures of cognitive function such as the *Mini-Mental Status Exam* (MMSE), Folstein et al. (1975) and the *Dementia Rating Scale* (DRS), Mattis (1976). The participants were also assessed with conventional neuropsychological tests like *Stroop Color-Word Interference Test, Trail-Making Test-B and Digit-span*. More complex tasks of executive function were assessed by the *Wisconsin Card Sorting Test*. Memory assessment was based on the *Rey Auditory Verbal Learning Test*. Visuoconstruction was assessed with tasks like *clock drawing*.

Assessment of mood and emotional state is a critical component of the evaluation of the MCI patient as emotional distress can cause or exacerbate cognitive problems. The assessment of mood was comprised of interview data and responses to brief self-report measures, such as the mini *Geriatric Depression Scale*. Neuropsychological scores for each population are presented in Table 1.

**Table 1:** **Means and SDs of demographics and general neuropsychological abilities**.

	**Older adults (*n* = 15)**	**aMCI patients (*n* = 25)**	**ANOVA/ANCOVAS**
**DEPRESSION**			
Mini GDS (cut-off < 2/5)	0.4 (0.9)	0.1 (0.3)	*F* < 1
**GLOBAL COGNITION**			
MMSE	28.9 (0.8)	26.7 (1.6)^*^	*F*_(2, 49)_ = 83.8^***^
DRS	21.9 (11.9)	42.9 (15.1)^*^	*F*_(2, 49)_ = 25.8^***^
**EXECUTIVE FUNCTIONS**			
WCST	18.8 (8.7)	25.1 (7.9)	*F*_(2, 49)_ = 8.2^**^
TMTB-A (s)	45.9 (13.4)	53.1 (24.1)	*F*_(2, 49)_ = 12.9^***^
Stroop	158.4 (119)	66 (48)	*F*_(2, 49)_ = 3.79^*^
Forward span	6.1 (0.3)	5.3 (1.3)^*^	*F*_(2, 49)_ = 14.9^***^
Backward span	3.6 (0.9)	2.9 (0.6)^*^	*F*_(2, 49)_ = 10.6^***^
**VERBAL MEMORY**			
Delayed recall	15.9 (0.25)	13.3 (1.6)^**^	*F*_(2, 49)_ = 33.9^***^
Total recall (3 trials)	45.8 (1.3)	32.7 (8.2)^***^	*F*_(2, 49)_ = 77.9^***^
Delayed total recall	15.9 (0.4)	11.6 (3)	*F*_(2, 49)_ = 95.1^***^
Intrusions	0	1.7 (1.4)^**^	*F*_(2, 49)_ = 28.1^***^
Perseverations	0	0.7 (1.5)	*F*_(2, 49)_ = 9.9^***^
Recognition: hits/false recognitions	15.8 (0.5)∕(0)	13.5 (1)∕1(1)	*F*_(2, 49)_ = 9.75^***^
			*F*_(2, 49)_ = 5.1^*^

* p < 0.05,

** p < 0.01,

*** *p < 0.001*.

As individuals age, they may experience changes in their auditory processing and/or cognitive abilities. In the present study, a neurologist performed the auditory test to assess the hearing level of both groups. Groups didn't differ significantly on the hearing level.

### Recordings

The standard auditory oddball paradigm was employed, as summarized below. Participants were engaged in a simple discrimination task. Two different tones were sequentially applied. The standard or non-target stimulus was appearing more often than the target stimulus. The series of tones was presented in randomized order, binaurally and at 70 dB sound pressure level (SPL) with a 10 ms rise/fall and a 100 ms plateau time. The standard (target) tone was set at 1 kHz (2 kHz) and corresponded to 80% (20%) of the stimuli, while the inter-stimulus time interval had been set as 2 s. The subject's task was to distinguish between the two tones by responding to the targets (via mentally counting them) and not responding to the rest stimuli. Participants had been instructed to pay attention in distinguishing the tones, count the target tones silently and report the total number at the end of the exam. Only subjects that “performed reasonably well,” had been included in the study. To this end, we included data from participant that reported a number of listened targets that deviated less than 3 from the actual number of delivered target tones. In addition, each subject was tested twice and the reproducibility of the averaged AERPs response waveform was examined (Näätänen et al., 1978).

EEG activity was recorded with a Neuropack 4 (Nihon-Kohden, Tokyo) equipment, after bandpass filtering within (0.1–50) Hz, with a sampling frequency *f*_s_ = 1024 Hz from scalp AgCl electrodes at Cz and Pz sites according to the 10/20 system referred to linked earlobe electrodes, with a right hand ground. Signals had been segmented into single-trial segments of 1 s duration, lasting from −100 ms to +900 ms with respect to stimulus onset. An on-line routine had automatically removed artifact contaminated trials based on extremely high amplitude levels. The recording was terminated as soon as a predetermined number of responses to target stimuli (30 trials) had been collected. The two types of trials (responses to target and non-target tones) were stored as distinct datasets for each subject. An additional artifact trimming step, based on the pattern analytic methodology of (Laskaris et al., 1997; Laskaris and Ioannides, 2001), was introduced so as to exclude any subtler outliers missed by the online routine. Additionally, we inspected visually the trials to further diminish any outlier missed by both the online routine and the pattern analytic methodology.

Electrophysiological activity was recorded from two different electrode positions at CZ and PZ but we analyzed the trials recorded from Pz. N100, N200, and P300 components are more prominent in PZ compared to CZ in an auditory oddball paradigm with counting process (Huang et al., 2011). Moreover, in general ERPs and the related components are measured mainly across the midline (FZ, CZ, PZ). Additionally, the N100, N200, and P300 are often measured mainly at the central (Fz, Cz, Pz) electrode sites with lateral electrodes typically not assessed, since the midline scalp distribution provides significant information about the attentional and mnestic processes thought to contribute to P300 generation (Donchin et al., 1986; Donchin and Coles, 1988; Picton, 1992; Johnson, 1993).

Mental counting in an auditory oddball paradigm is a demanding task that needs various resources in order to be completed like concentration, attention, perception, cognition, and memory. The functional abilities and generators of N100, N200, and P300 in the brain have been reported. N100 is involved in general attention, and its generator is regarded as the primary auditory cortex (Hillyard et al., 1973; Kaga et al., 2004) where the most closest sensor in the midline closed to temporal lobe is Pz. P300 is involved in selective attention or cognitive ability, and its generators are regarded to be the hippocampus or limbic system and cerebral cortex (Donchin et al., 1975, 1986; Halgren et al., 1980; Donchin and Coles, 1988). N200 is involved in pre-attentive detection and superimposed with mismatch negativity and it is a faster component that reflects cognitive processes of stimulus evaluation, selective attention and conscious discrimination (Patel and Azzam, 2005).

## Methods

In contemporary neurosciences, the various patterns of oscillatory activity are considered as signatures of the cortical networks and key players in brain function by shaping the dynamic substrate of perception, memory, and consciousness. The oscillatory coupling between distinct neuronal assemblies is postulated as a principal mechanism for information exchange and integration (Varela et al., 2001; Buzsáki and Draguhn, 2004; Buzsaki, 2006). Cross-frequency coupling has recently been established as an additional communication channel and provided a novel perspective for characterizing and understanding the long established system of brain oscillations (Canolty and Knight, 2010). Among the possible CFC mechanisms, phase-amplitude coupling (PAC) is the one most commonly encountered in experimental brain research. Here, PAC is examined among the following 7 brain rhythm, {δ, θ, α_1_, α_2_, β_1_, β_2_, γ}, defined respectively within the ranges {2–4 Hz; 4–8 Hz; 8–10Hz; 10–13Hz 13–20 Hz; 20–30Hz, 30–45Hz}. Among the available PAC estimators, we adopted the one based on the phase coherence measure (Cohen, 2008; Voytek et al., 2010) and further adapted it so as to operate across trials and provide time-resolved profiles of CFC that would be studied in relation with the established components of the cognitive response (N2, P300, SW). In the followings, we first introduce the PAC estimator. Then, we describe its “ensemble” operation mode that is denoted, hereafter, as time-varying PAC (^TV^PAC). Finally, we outline the machine-learning strategy employed for identifying the PAC-features with the highest discriminatory power for aMCI detection. The latter methodological step is an important ingredient of this work, since our data-driven approach resulted in a multitude of PAC measurements, parameterized by the (possibly-interacting) pair of brain rhythms and the corresponding time-interval (that the particular functional interactions occurred). To derive the oscillatory activity of each brain rhythm, a 3rd order Butterworth filter was applied, in zero-phase mode, to concatenate multi-trial responses. After filtering, a segmentation into filtered single-trial response was performed.

### PAC estimation: the basic algorithm

Described in a more generic setting, let x(*t*), *t* = 1, 2, …, T is the recorded single-sensor signal at hand. Based on filtered versions of this signal, cross-frequency interactions will be sought complying with a form in which the phase of low-frequency (LF) oscillations modulates the amplitude of high-frequency (HF) oscillations. Using narrowband filtering, the two signals x_LF_(*t*) and x_HF_(*t*) are first formed and, then, their complex analytic representations z_LF_(*t*) and z_HF_(*t*) are derived by means of Hilbert transform (HT[.]).

$${\text{z}}_{\text{LF}}(\text{t})=\text{HT}[{\text{x}}_{\text{LF}}(\text{t})]\;=\;\left|{\text{z}}_{\text{LF}}(\text{t})\right|{\text{e}}^{\text{i}\phi_{\text{LF}}(t)}\;=\;A_{\text{LF}}(\text{t}){\text{e}}^{\text{i}\phi_{\text{LF}}(t)}\;,$$

$${\text{z}}_{\text{HF}}(\text{t})=\text{HT}[{\text{x}}_{\text{HF}}(\text{t})]\text{ = }\left|{\text{z}}_{\text{HF}}(\text{t})\right|{\text{e}}^{\text{i}\phi_{\text{HF}}(t)}\text{ = }A_{\text{HF}}(\text{t}){\text{e}}^{\text{i}\phi_{\text{HF}}(t)}$$

In this way the amplitude and phase dynamics, captured respectively by the envelope A(*t*) and instantaneous phase ϕ(*t*) signal, can be treated independently. Next, the envelope of the higher-frequency oscillations A_HF_(*t*) is bandpass-filtered within the range of LF oscillations and the resulting signal undergoes an additional step of Hilbert transform so as to isolate its phase-dynamics component ϕ′(*t*),

$$z^{\prime }(t)=HT[A_{HF,\;LF}(t)]=\left|z^{\prime }(t)\right|e^{i{\phi^{\prime }}_{HF}(t)}=\;\left|z^{\prime }(t)\right|e^{i\phi_{LF\to HF}(t)}$$

that reflects the modulation of HF-oscillations amplitude by the phase of the LF-oscillations. The corresponding timeseries will be used to estimate PAC, by means of phase-locking (or synchronization index) technique.

(1) $$PLV(LF,HF)=PLV_{LF\to HF}=\left|\frac{1}{T}\sum_{t=1}^{T}e^{i(\phi_{LF}(t)-{\phi^{\prime }}_{HF}(t))}\right|$$

Phase-locking value PLV ranges between 0 and 1, with higher values indicating stronger PAC interactions (i.e., higher comodulations).

**Figure 1:**
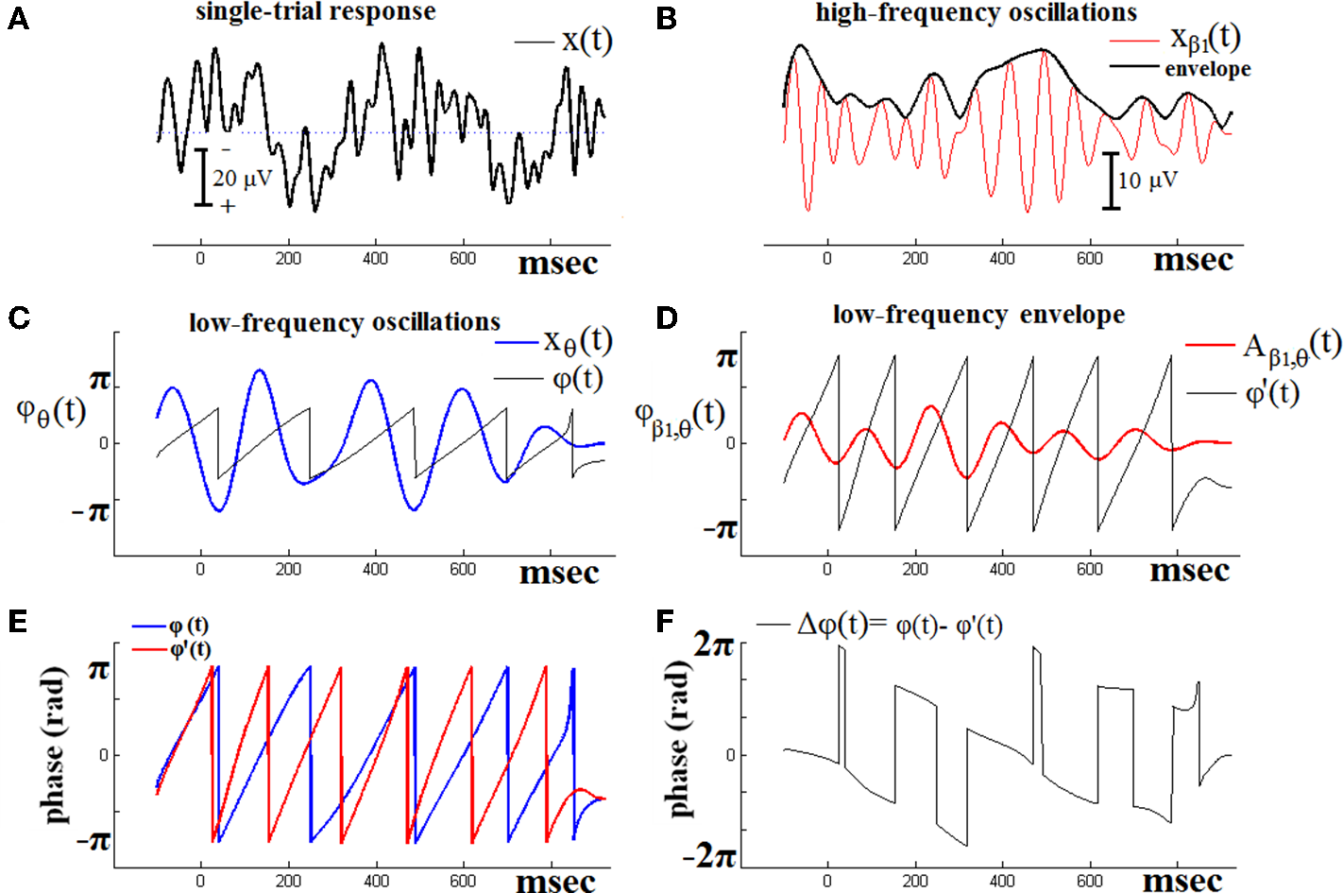
**The algorithmic steps for PAC estimation**. Using the first single-trial signal **(A)**, from the cognitive responses of a control subject, we demonstrate the detection of coupling between θ and β_1_ rhythm. To estimate θ-β_1_ PAC, the raw signal was band-pass filtered into both a **(B)** low-frequency θ (4–8 Hz) component where its envelope is extracted as well as **(C)** a high-frequency β_1_ (13–20 Hz) component where its instantaneous phase is extracted. **(D)** We then extracted the amplitude and the instantaneous phase of the band-passed β_1_ (13–20 Hz) and filtered this amplitude time series at the same frequency as θ (4–8 Hz), giving us the θ modulation in lower β amplitude. **(E)** We then extracted the instantaneous phase of both the θ-filtered signal and the θ-filtered lower-β amplitude and computed the phase-locking between these two signals. The latency depended differences **(F)**, will be used in estimating the phase-locking that will reflect the PAC-interaction between the two involved brain rhythms. This phase-locking represents the degree to which the lower β (β_1_) amplitude is comodulated with the θ phase.

Figure 1 demonstrates the previous algorithmic steps using a single-trial ERPs signal from one of the NI subjects. PAC interactions are examined, between LF oscillatory response activations corresponding to θ brain rhythm and HF activations corresponding to β_1_ rhythm. The original signal is shown in Figure 1A. The HF version of this signal is depicted in Figure 1B, along with its envelope. Just beneath (Figure 1D), is shown the low-pass filtered (within θ frequency range) version of the previous envelope [i.e., the A_β1_, θ(*t*) signal]. The overriding, saw-like, trace corresponds to its instantaneous phases $\phi_{\beta 1}^{\prime }$(*t*). On the other hand, the LF version of the original signal is depicted in Figure 1C, along with the trace of the corresponding instantaneous phases ϕ_θ_(*t*). The ϕ_θ_(*t*) and ϕ'_β1_(*t*) traces have been plot aligned in Figure 1E, so as to form the instantaneous phase differences as shown in Figure 1F. It is this sequence of phase-differences Δϕ(*t*) that enters in Equation (1) and will be “integrated” across time via averaging the corresponding directional vectors e^*i*Δϕ(*t*)^ in the complex domain. It becomes clear that the length T of this sequence has to be long enough, so as the PLV index to result into a reliable measure of PAC.

### Across-trials PAC estimation: the ^TV^PAC estimator

To provide a time resolved PAC profile, that would incorporate the event-related CFC interactions which occurred systematically during the AERPs experiment, we invoked the standard algorithmic strategy for estimating the timecourse of PLV in multi-trial datasets. All the above mentioned steps leading to the time-integration in eq(1), were performed for every single-trial response x_*j*_(t), *j* = 1,…, N available for each subject.

Next, the set of derived phase-differences Δϕ_*j*_(*t*) was formed and, finally, across-trial “integration” was performed.

(2) $${}^{TV}PLV_{LF\to HF}(t)=\left|\frac{1}{N}\sum_{j=1}^{N}e^{i\Delta \phi_{j}(t)}\right|$$

This resulted in a PAC-trace that had the same temporal resolution as the original single-trials. Considering the low number of trials available for each participant (on average 27 trials), we decided to use a temporal window of 2w + 1 samples and extend the integration in extracted segments around each latency.

(3) $${}^{TV}PLV_{LF\to HF}(t^{\prime })=\left|\frac{1}{N(2w+1)}\sum_{t^{\prime }=t-w}^{t+w}\;\;\sum_{j=1}^{N}e^{i\Delta \phi_{j}(t^{\prime })}\right|$$

With the scope of avoiding redundancies, the above computations were performed by means of a stepping window (with no overlaps between successive segments). In the above equation, this is implied by the time index t', that runs over the number of formed segments. Hence, the derived ^TV^PAC-traces were of reduced temporal resolution, so as to smooth out unduly variations (in particular, a window corresponding to 20 ms had been employed).

**Figure 2:**
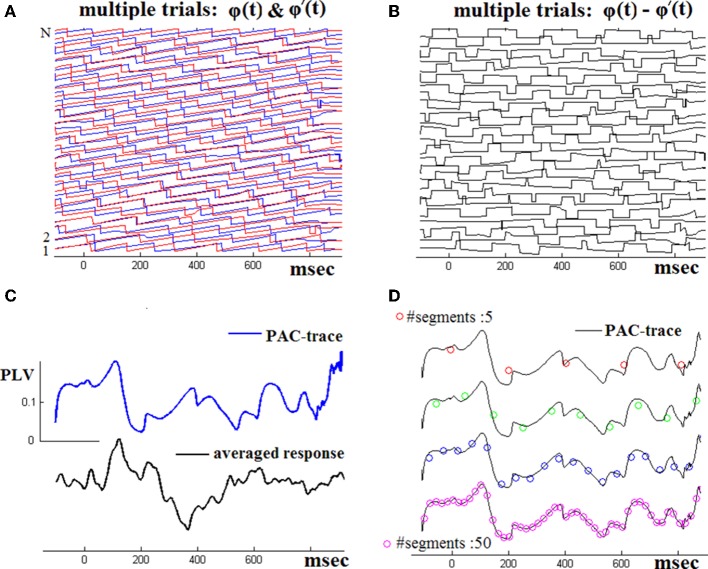
**Across-trials PAC-estimation**. By repeating the steps shown in the previous figure, the instantaneous phase differences for the whole set of single-trials have been computed **(A,B)**. The ^TV^PAC trace [reflecting PLV(*t*) measurements for θ → β_1_ interaction], at full temporal resolution, is shown **(C)**, together with the ensemble average waveform (from the wideband signals). ^TV^PAC traces from a stepping window (of various widths) are shown in **D**).

**Figure 3:**
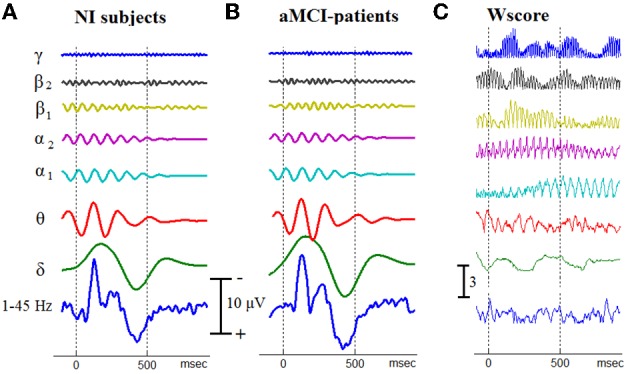
**An approximation of the temporal patterning of oscillatory cognitive responses by means of Grand Averaging**. The GA-traces for Non-impaired controls **(A)**, and aMCI patients **(B)**, have been derived independently for each brain rhythm. Using the corresponding temporal patterns from all the 40 participants, the separability between aMCI patients and NI controls has been measured at every latency and for each brain rhythm. A common scale is used for all the traces within the same stack. **(C)** To demonstrate the temporal separability of oscillatory responses, Wscore is presented for each individual brain rhythm.

Figure 2 demonstrates the operation of the employed ^TV^PAC estimator using (in continuation of Figure 1) the whole set of single-trial responses of the NI subject. The two panels in first row visualize the computed instantaneous phases of θ-rhythm and β1-envelope-related oscillations and the subsequently derived phase differences Δϕ_*j*_(*t*). The ^TV^PLV(*t*) timeseries computed via Equation (2) is shown in Figure 3C. Smoother estimations of latency-dependent PAC, computed via Equation (3) at varying resolution, are provided in Figure 2D. To ease comparison, the corresponding ^TV^PLV(*t*′) sequences have been relatively shifted along y-axis and superimposed on the full-resolution PAC waveform.

### Handling ^TV^PAC measurements and identifying discriminative events

For each subject independently the ^TV^PAC profiles were computed for all possible pairs among the 7 defined brain rhythms. Hence, there were in total ½ × 7 × 6 = 21 (LF → HF) PAC-related sequences. Each sequence consisted of 50 measurements and stored in a vector, the dimensions of which corresponded to distinct latencies (i.e., the temporal segments they had come from). The basic form of handling all PLV-measurements was this of a [21 × 50] matrix for each subject. An alternative form to represent these measurements was by means of a 3D array of size [7 × 7 × 50], which corresponded to a time series of connectivity patterns (or graphs). That was less economical in terms of memory storage (as the entries corresponding to HF → LF interactions had been zeroed), but conceptually more fruitful in terms of presentation. With that approach, it become possible to visualize multiple cross-frequency interactions at “latencies-of-interest” and detect emergent event-related PAC patterns (for instance see **Figure 6**).

To systematize the comparison between aMCI patients and NI subjects, the ^TV^PAC measurements were considered as the initial set of extracted features, based on which the classification should be performed and a “filtering” scheme for selecting the most useful among them was applied. The overall scheme was based on matlab routine *rankfeatures* (with the “Wilcoxon” criterion activated), that realized feature ordering based on a score measuring class-separability. In more details, for each pair of interacting frequencies and every latency the corresponding PLV-measurements for both groups ({^aMCI#^PLV_LF, HF_ (*t*′)}_*i* = 1:25_, {^NI#^PLV_LF, HF_ *(t*′*)*}_i = 1:15_) were gathered as two distinct sets of scalars and then statistically compared by means of wilcoxon rank-sum test that resulted in a score, denoted as Wscore, that its higher values indicated more deviating distributions. The use of wilcoxon test was motivated by its non-parametric nature and established robustness. The main idea was that the selection of important features for aMCI detection would be accomplished by keeping the most discriminative ones; in other words, the top-ranked entries from the [21 × 50] PLV-matrix of each participant. The battery of selected PAC measurements would form a composite multifaceted set of feature, based on which a biomarker would be built by way of a popular multivariate classifier.

Considering the small-sized sample situation of our study (only 40 participants were included) and the danger of “overfitting,” since we were obliged to optimize feature-selection and then design a classifier using the same dataset, we decide to introduce an additional step of bootstrapping (Hastie et al., 2003). During that step, by sampling (with replacement) from the available set of 40 PLV matrices, we built 1000 equal-sized datasets and repeated the estimation of Wscore for each (LF, HF) pair and latency *t*′. The consistency of each PLV-related feature was measured by the following index:

(4) $$Wscore^{*}=Wscore^{*}(PLV_{LV\to HF}(t^{\prime }))=\frac{\bar{Wscore_{bootstraps}}}{std(Wscore_{bootstraps})}$$

Wscore^*^ reflected (inversely) the *coefficient of variation* for the measurements over the bootstrap samples and served as a refined score for ranking the PAC-estimates. In that way, the reported results (selected frequency pairs and latencies) enjoyed a power that was not limited in the particular cohort of 40 participants. More importantly, the corresponding bias during the subsequent stage of designing the classifier (based on the selected top-ranked features) was reduced.

## Results

In this section, apart from the main results concerning the introduced biomarker, we have included some additional indicative results in order to provide further justification of employing PAC in analysing AERPs responses and appreciate its dynamic character. In what follows, only signals recorded at Pz electrode have been considered. The section begins with the presentation of the Grand-Averaged responses from the two groups, so as to provide an indication about the difficulty of the problem of discrimination between aMCI patients and NI-controls based on the temporal patterning of the (supposedly) time-locked responses. Then, the emergence of PAC as a prominent characteristic of the underlying cognitive processes is demonstrated, by comparing PAC measurements from responses to target and non-target stimuli. Next, aMCI is shown to be accompanied by aberrations in the response-related mechanisms of CFC. Then the design of biomarker using relevant PAC-features is presented and its performance is evaluated. Finally, some quantitative comparisons using popular alternative descriptors are provided.

### The event-related oscillations and grand averaged responses

After filtering the single-trial responses within the frequency bands defined for the examined brain rhythms, a set of 7 temporal patterns was obtained, for each participant, via ensemble averaging. Figure 3, in the first two columns, presents the corresponding within-group averaged responses in a stack-plot format. In addition, the corresponding wide-band patterns (after band-pass filtering of single-trials within 1–45 Hz) have been appended at the bottom of each stack, making easier the identification of the main morphological components of AERPs, namely N100, N200, P300, and SW (slow-wave component). It becomes apparent that the discrimination between aMCI patients and NI subjects, based on the patterning of (averaged) cognitive responses, is not an easy to perform task. To express such a trend in quantitative terms (that in addition would facilitate comparisons among the various rhythms), we considered these temporal patterns as set of distinct features extracted from each subject (in total 8192 = [(7+1) bands × 1024 latencies]) and used the wilcoxon score to measure its potentiality for aMCI detection. The rightmost column in Figure 3 presents the computed Wscore-measurements in a format fully compatible with the associated temporal patterns. It is evident that each oscillatory component shows its own idiosyncrasies which predominantly reflect the corresponding characteristic timescale. Furthermore, some morphological components appear as coupled (i.e., time-locked) with the discriminability of some particular oscillatory components (for instance SW component coincides with an increase/decrease in discriminability of α_1_/α_2_ rhythm). Interestingly, the γ_1_ rhythm is associated with the higher Wscores, the temporal profile of which shows a clear modulation from a slower oscillation rhythm. Finally, it is worth noting that the discriminabilty in the wide-band filtered signals (see the Wscore profile at the bottom) is much lower than in the predefined brain rhythms.

### Contrasting ^TV^PAC measurements from evoked and event-related responses

The first step toward the construction of a PAC-related biomarker was to establish that the probed CFC phenomena could be, indeed, associated with the examined cognitive processes of the subject performed the auditory discrimination task. The inherent experimental design (AERPs, which by-default includes two comparable stimulation conditions and requires context-related response), offered a unique opportunity to test that. We systematically compared the ^TV^PAC estimates obtained from trials, in which a non-target stimulus had been delivered, with the corresponding estimates from trials required from the participant to perform the cognitive task (i.e., detection and mental counting).

**Figure 4:**
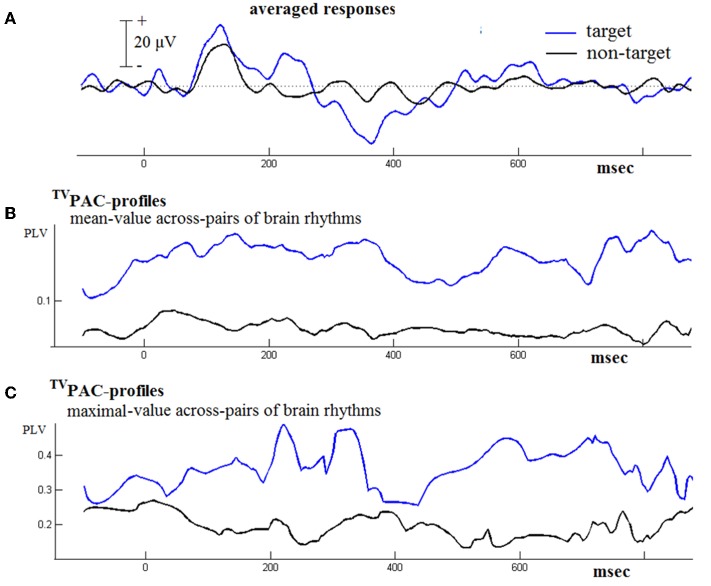
**Contrasting the ^TV^PAC profiles from the responses (of an NI subject) to target and non-target stimuli. (A)** The averaged evoked/event-related response is shown in black/blue. **(B)** A rough summary of ^TV^PAC measurements estimated by means of averaging across all frequency-pairs (LF, HF), independently for each latency. **(C)** A summarizing profile derived by keeping the maximum PLV value at every latency.

Figure 4 contains results from such a comparison based on the responses from a NI-subject. Firstly, it needs to be mentioned here, that during that contrasting-process two distinct time series of CFC connectivity patterns were originally encountered. In order to facilitate visualization, we employed the following steps. The whole set of latency-dependent PLV-values, which had been obtained at full temporal resolution using Equation (2), was formatted as a 2D matrix of [#_frequency-pairs_ × #_latencies_] = [21 × 1024] size. At every latency *t*, the mean and the maximum of the corresponding 21 PLVs were computed. The resultant temporal-profiles of cross-frequency interactions have been included in the middle and bottom panel of Figure 4. They provide only rough summaries (of the multitude) of interaction happening during the physical reaction to stimulus and its subsequent evaluation. However, by contrasting them between evoked and cognitive responses it becomes clear that an increased CFC can be associated with the cognitive aspects of response. A similar behavior was observed in the data from other normal subjects well (not shown here). In particular, the profile of maximal instantaneous PLVs reflected a waxing and waning behavior that made necessary the disentanglement of cross-frequency interaction, carried out as described below.

**Figure 5:**
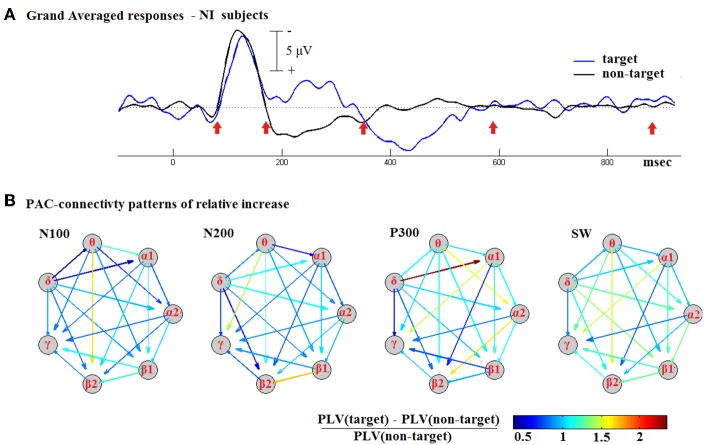
**Contrasting the CFC during responses to target and non-target stimuli based on group-averaged data from NI subjects. (A)** The N100, N200, P300, and SW deflections were first detected in the waveform of Grand-Averaged ERPs response. **(B)** Using the corresponding temporal segments, PAC-related connectivity snapshots were then derived, for target and non-target stimuli separately, and then used to express the relative increase in coupling. A common color scale was used across for all shown graphs.

The ^TV^PAC measurements for all NI subjects were first assembled and then averaged on a latency-by-latency basis. Two timeseries of connectivity patterns were formed, representing the dynamics of CFC coupling during the response to target and non-target stimuli. They were treated as 3D tensors, and denoted respectively as ^target^**GA_PLV** and ^non-target^**GA_PLV**. Based on the grand-averaged responses, we identified the latency-intervals, shown in Figure 5A, that corresponded to the morphological components of cognitive responses. By means of integration within the denoted temporal segments, we estimated a pair of CFC-patterns roughly corresponding to the identified components (deflections). We then formed the pattern of relative increase for each deflection (N100, P200, P300, and SW).

(5) $$\begin{array}{l} Relative-Increase\;(deflection)=\\ \;\frac{\sum_{segment}^{target}GA\_PLV_{LF\to HF}(t)-\sum_{segment}^{non-target}GA\_PLV_{LF\to HF}(t)}{\sum_{segment}^{non\text{-}target}GA\_PLV_{LF\to HF}(t)} \end{array}$$

The Relative-Increase pattern consisted of 21 PLV-values indicating the influence of cognitive task in the PAC-coupling during the time-interval associated with each one of the main AERPs deflections.

Figure 5B includes the derived patterns in the form of weighted directed graphs: the nodes correspond to brain rhythms and the edges to estimated levels of PAC. The color in each arrow reflects the weight of the corresponding edge, that is the Relative-Increase in the strength of LF → HF interaction. The depicted snapshots of functional connectivity strengthening are clearly suggestive of a positive correlation between CFC and the cognitive task. The strongest increase emerged during the P300 deflection and was associated with a δ → α_1_ interaction.

### Contrasting ^TV^PAC measurements from aMCI-patients and NI-subjects

**Figure 6:**
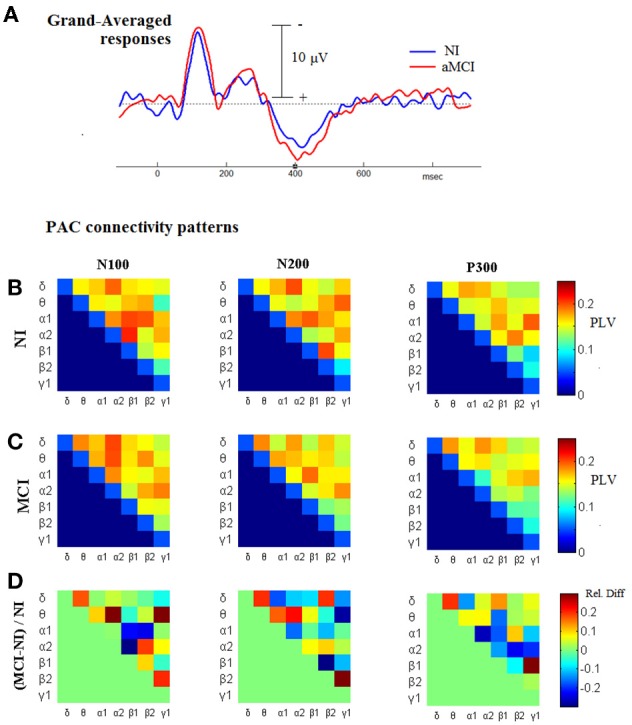
**Comparing the level of CFC in cognitive responses, between NI and aMCI participants. (A)** Grand-Averaged waveforms from the cognitive responses (AERPs) of both groups. **(B,C)** Group-related (grand-averaged) PAC-connectivity patterns for the temporal-segments corresponding to N100, N200, and P300 deflections. **(D)** The corresponding patterns of relative differences, derived so as to express deviation from normality; red/blue indicates higher/lower PAC levels in MCI subjects relatively to NI subjects.

Considering the previous observation about the increased CFC associated with cognitive responses, and as the next step before introducing the CFC-related biomarker, we proceeded by comparing the ^TV^PAC measurements between the patients and the healthy controls, based on their responses to target stimuli. From the corresponding 3D tensors of the group-averaged measurements, ^aMCI^**GA_PLV** and ^NI^**GA_PLV**, we derived patterns of cross-frequency coupling that were associated with the latency-range of the three main deflections in the Group-averaged waveforms (N100, N200, P300; as shown in Figure 6A). These connectivity snapshots have been presented in tabular format for NI and aMCI groups, respectively, in the first and second row of Figure 6B. The patterns in the last row are reflecting the contrast between the paired-patterns, which has been formulated as a relative difference expressing departure from normal behavior.

(6) $$\begin{array}{l} Relative\text{-}difference\;(deflection)=\\ \;\frac{\sum_{segment}^{MCI}GA\_PLV_{LF\to HF}(t)-\sum_{segment}^{NI}GA\_PLV_{LF\to HF}(t)}{\sum_{segment}^{NI}GA\_PLV_{LF\to HF}(t)} \end{array}$$

The most important observation, that can be made based on Figure 6, is that the CFC differences are neither unidirectional nor stable across the different segments. Hence, it is not a straightforward task to craft a biomarker based on ^TV^PAC-measurements. Despite this complexity, the level of relative-difference approaches the 30% (in both directions), which is much higher than the corresponding difference in the morphology of grand-averaged responses (shown in Figure 6A).

### The CFC-Biomarker: insights into the ^TV^PAC features

**Figure 7:**
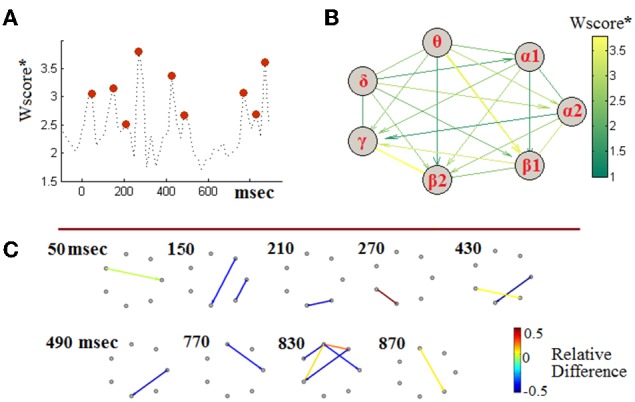
**Identifying discriminative PAC-interactions (group-level analysis). (A)** The temporal profile of the (quasi-instantaneous) maximal separability measure and the identification of the timing of most discriminative PAC couplings. The 9 red discs indicate the local maxima in the timecourse. **(B)** A graphical representation of the maximal PAC-couplings (stacked across time). **(C)** Snapshots of differences between grand-averaged PAC-patterns, at instances of high discriminability. The shown graphs correspond to the 9 segments detected in **(A)**. Positive/negative values of Relative-Difference indicate higher/lower PAC for the MCI participants relatively to NI participants. To enhance visibility, edges associated with a Wscore^*^ lower than 2 are not shown.

Taking into account the observed non-stationarities, we attempted to define a single, though composite, biomarker that could encompass the complex dynamics of cross-frequency interactions for the sake aMCI detection. Following the machine-learning methodology described in Section Handling TVPAC Measurements and Identifying Discriminative Events, we first identified the most discriminative instances of CFC along with the participating brain rhythms. Toward this end, we first derived a 3D tensor of Wscore^*^-values. That tensor was of a size equal with the size of PLV-related tensors (i.e., [7 × 7 × 50]). The entries of the tensors contained the separability between aMCI and NI participants and were parameterized by the interacting frequencies and the temporal segments. To gain some insights into that set of measurements, we identified, independently for each segment, the maximal PLV-value (among the 21 included in the corresponding connectivity snapshot). The obtained temporal profile has been included in Figure 7A, providing additional evidence about the dynamic nature of the PAC-phenomena and the way they deviate between healthy and impaired cognition. This temporal perspective was complemented by an interaction-pattern perspective, which was derived by estimating the maximum PLV-value across time (i.e., among the set of corresponding 50 values) independently for each LF → HF interaction. The emergent pattern has been included, as a weighted directed graph, in Figure 7B, with edges colored in proportion to the Wscore^*^. From this graphical synopsis of interactions between brain rhythms, two interactions appear to stand-up, namely a θ → β_1_ and β_2_ → γ. It is necessary to mention here, that in the particular visualization the interactions have been scored according to their importance in the particular task of discriminating between aMCI-patients and NI-subjects, without providing any hint about the “loss” or “gain” in CFC-strength due to cognitive impairment. For this reason, we accompanied the above two perspectives with the additional one of evolving-graphs (Figure 7C). Using the patterns of relative-difference (derived in analogy with Equation 6, but based on particular segments), from the segments corresponding to the 9 maxima in Figure 7A (indicated via red discs), we have drawn a sequence of connectivity patterns, in which the edge color indicates the relative-difference and, also, its sign. From this timeseries, we can specify that the above mentioned θ → β_1_ modulation corresponds to the latencies of SW deflection, while the β_2_ → γ modulation corresponds to the latencies of N200 deflection (see Figure 6A). Both interactions exhibit increased strength in the case of aMCI subjects. However, the set of most discriminative interactions includes also interactions showing the converse trend. One should notice that the level of estimated relative-differences, now, ranges from 50% decrease to 120% increase and compare it with the level shown in Figure 6B, which corresponded to lower-resolution analysis (segments of morphological components).

### The CFC-Biomarker: design and performance measures

The comparative study between the two groups of participants revealed that in order to fully exploit the ^TV^PAC measurements, for the purpose of aMCI detection, it was mandatory to resort to a learning machine. The framework of multivariate classifiers was appropriate for incorporating the derived PLV-values and implementing the discrimination task (aMCI-patients vs. NI-subjects). Since the emphasis of this work was put on *feature-engineering* (Bengio et al., 2013), we employed two widely-used classifiers and refrained from further improving aMCI detection by means of a more sophisticated classifier. We first experimented with the k-nearest-neighbor classifier and then with the ***linear support vector machine*** (**SVM**). Since the obtained results were slightly better for the latter learning machine, we decided to confine the following presentation accordingly.

The feature-vectors (FVs) used as input to the SVM consisted of the 30 most discriminative ^TV^PAC-related characteristics, as identified from the feature-ordering step, which was based on Wscore^*^. The reader can refer to the visualization of Figure 7C, where the most informative among the candidate characteristics have been included. The dimensionality *p* = 30 of the FVs was set experimentally. Starting with the two most discriminative PAC-characteristics, namely the PLV_θ → β1_ (870 ms) and PLV_β2 → γ_ (270 ms), we continued to include features according to their ranks and measuring the performance of the SVM classifier. The classifier peaked its performance after including the 30th characteristic and kept behaving equivalently till the 50th one.

The introduced biomarker was realized via feeding the 30D FVs to the *svmtrain* and *svmclassify* functions from the *statistics and machine learning toolbox* of MATLAB. To reliably estimate its performance in the task of aMCI detection, we employed two alternative validation schemes, which assessed how the biomarker would perform to an independent data set of AERP-responses. The first scheme was the *leave-one-out cross-validation* (**LOOCV**). Each subject, in turn, was considered of unknown classification. By using his FV, a diagnosis (impaired or healthy) was attempted, through the SVM classifier. The classifier had been previously trained using the FVs of the rest of 39 subjects, who had been considered of known classification. By comparing the SVM predictions with the correct labels, we estimated classification accuracy and in addition the sensitivity and specificity of the introduced biomarker. The second scheme was a two-fold cross-validation (**2-CV**), that was repeatedly applied as follows. We randomly picked a group of 35 subjects, for whom the classification labels were considered to be known (training-set). The remaining 5 subjects formed the second fold, for whom the classification labels were considered unknown. An SVM was first trained based on the FVs of participants in the training set and, then, used to predict the classification-labels of the participants in the test-set. The SVM predictions were used to assess the performance. The whole procedure was repeated 200 times and the mean values of accuracy, sensitivity and specificity were finally reported. Table 2 includes the results from both validation schemes.

**Table 2:** **Biomarker performance in aMCI detection—SVM operating on ^TV^PAC characteristics**.

**%**	**LOOCV**	**2-CV**
**Accuracy**	97.5	95.0
**Sensitivity**	100.0	96.0
**Specificity**	93.3	93.0

### Comparing with alternative representations

As the very last part of this study, we applied the overall machine-learning methodology to representations of the cognitive responses obtained via diverse methodological approaches. Starting with an initial set of extracted characteristics, we ranked its elements according to the Wscore^*^-index and selected the most discriminative ones as the set of FVs to be fed into a linear SVM so as to achieve the classification between aMCI-patients and NI-controls. The achieved classification performance was expressed as in the case of the introduced ^TV^PAC-based biomarker.

The first utilized representation was based on *time-locked averaging* (TLA), and included the set of 7 temporal waveforms (one for each brain rhythm) as extracted characteristics (7 × 1024). It encapsulated the temporal patterning of cognitive response and was well-aligned with the conventional (averaged) format, that these responses are encountered in clinical practice. The second examined representation was based on *short-time Fourier transform* (STFT) and included the averaged (across-trials) spectrogram. That representation is generally considered suitable for incorporating the spectro-temporal profiles of event-related induced oscillations. Finally, as an alternative representation suitable for incorporating the multi-scale character and the non-stationarities of the response, the averaged scalogram derived via *Morlet wavelet transform* (WT) was examined. The scoring of the involved characteristics in the case of TLA can be seen in Figure 3C. The relevant scoring corresponding to the two transforms has been included as Supplementary Material. Table 3 presents, in comparative fashion, the accuracy of all the potential biomarkers as this was assessed, via cross-validation, based on the available data. It should be noticed here that the final number of selected features had been optimized independently for each approach (as described is Section The CFC-Biomarker: Design and Performance Measures). From Table 3, the superiority of the introduced representation (compare first row with the rest ones) becomes evident.

**Table 3:** **Comparing representations based on the performance of SVM-based aMCI detection**.

**Accuracy (%)**	**LOOCV**	**2-CV**
**^TV^PAC**	97.5	95.0
**TLA**	70	63.5
**STFT**	62.5	64.4
**WT**	77.5	75.5

## Discussion

A novel connectomic biomarker for detecting aMCI was introduced, based on time-resolved estimates of cross-frequency coupling estimates from single-trial cognitive responses recorded during an ordinary auditory oddball paradigm. It is based on a multiparametric signature of cognitive processes and reflects the complex dynamical interactions among brain rhythms that take place during the stimulus evaluation. Our experimentations showed a high classification rate (95%) based on the proposed ^TV^PAC features. In addition, the superiority of our approach against alternative popular methodologies was demonstrated by bringing them within the same learning framework (see Table 3). The novel concept of dynamic CFC during AERPs response is added to the available EEG-related diagnostic tests for cognitive impairment (Henderson et al., 2006; Lehmann et al., 2007; Abásolo et al., 2008; Dauwels et al., 2010; Laskaris et al., 2012; Latchoumane et al., 2012; Fraga et al., 2013; Tarnanas et al., 2014a, 2015b).

EEG signals are nonlinear and non-stationary signals and contain oscillatory activity generated by different cortical areas. To understand the interactions between brain rhythms of different frequency content, EEG signals should be studied in terms of CFC (Canolty and Knight, 2010). There are four main types of CFC as documented in (Jensen and Colgin, 2007): (i) power to power, (ii) phase to phase, (iii) phase to frequency, and (iv) phase to power. There is accumulating evidence that the last form of CFC, the so- called phase-amplitude modulation-coupling (PAC), occurs very often (Cohen, 2008; Osipova et al., 2008; Tort et al., 2008, 2009, 2010; Cohen, 2008; Cohen et al., 2009a,b; Colgin et al., 2009; Axmacher et al., 2010a,b; Voytek et al., 2010). It is hypothesized that CFC between different frequency bands within and between sensors is the key mechanism for the integration of both local and global processes and hence being related to the uninterrupted communication between different brain states expressed within a characteristic frequency band (Canolty and Knight, 2010; Buzsáki and Watson, 2012).

The pivotal role of CFC in neuronal computation, communication and learning has been recently demonstrated. In particular, the strength of PAC differs within and across brain areas in relation to task, changes rapidly in response to a stimulus (visual and auditory or both), motor and cognitive events and (anti)-correlates with performance during learning tasks (Canolty and Knight, 2010). Thus, CFC might serve as a key mechanism of a syntactical organization of communication between brain areas that oscillate on a prominent frequency characteristic of a specific cognitive function. Phase orchestrates such communication, while the interacting direction (toward the amplitude of a higher frequency rhythm) further supports the idea of hierarchical cross-frequency coupling organization (Buzsáki and Watson, 2012). In a recent study, based on normal aging and a short-term memory task, CFC unfolded the inefficient organization of competing brain networks and finally indicated the neural mechanism which is responsible for this integration breakdown (Pinal et al., 2015).

PAC phenomena, often mentioned as “nested oscillations,” occur when the amplitude of an oscillation at a particular frequency is modulated by the phase of a lower frequency oscillation. This form of CFC has been suggested as the key mechanism for, amongst many others significant cognitive functions, working memory (Jensen and Lisman, 1998), spatial exploration (Lisman and Buzsaki, 2008), and visual perception (VanRullen and Koch, 2003; Palva and Palva, 2011). Moreover, it is the cross-frequency coupling between different frequency bands that has been hypothesized to be the carrier mechanism for the interaction of local and global processes and hence being directly related to the integration of distributed information (Jirsa and Müller, 2013).

The proposed biomarker exploits the dynamic behavior of the phase-to-amplitude coupling (PAC) between frequency pairs (Canolty and Knight, 2010; Voytek et al., 2010; Jirsa and Müller, 2013). There are various indications about neural oscillations interacting in a time-varying manner (Buzsáki and Draguhn, 2004; Buzsaki, 2006; Buzsáki and Watson, 2012). Neural oscillations reflect interactions between the time (phase) and the amplitude of oscillatory activity of individual components captured even from a single sensor. Task-relevant oscillations of different frequency component recorded at a single sensor reflect different cognitive functions related to specific local brain areas. Studying CFC in a dynamic fashion while subjects performed a task is of significant importance. It is well-known that cortical frequency ranges can form temporal windows in neural dynamics (Canolty and Knight, 2010; Buzsáki and Watson, 2012) where the phase of a lower-frequency band can modulate the amplitude (power) of a higher frequency. In quasi-stable temporal windows, this form of communication via PAC can be expressed with different frequency pairs which interact accordingly to the demands of the task and the cognitive resources that should be accessed to perform the task and to process the external stimuli and in general the task.

The scope of this work is to introduce a reliable dynamic connectomic biomarker (DCB) for the detection of abnormal cognitive declinement due to MCI. To address the prominent non-stationarity of ERP functional connectivity and the hierarchical organization of brain rhythms, the adaptation of a dynamic functional connectivity approach (Dimitriadis et al., 2010b, 2012a,b, 2013a,b, 2015b; Ioannides et al., 2012; Kopell et al., 2014) based on CFC (Canolty and Knight, 2010; Buzsáki and Watson, 2012; Dimitriadis et al., 2015a) is necessary. The predictive power of the proposed (TICB) was 95% (Dimitriadis, 2015c) and it is the first TICB based on CFC biomarker in relation to a brain disease compared to various connectomic biomarkers extracted from static graphs (see reviews Sporns, 2014; Stam, 2014; Braun et al., 2015). A recent study explored cross-frequency modulations and revealed a disappearance of δ modulations of β frequency band and an appearance of δ modulations in the θ frequency band, both intensified by the severity of the disease (Fraga et al., 2013).

Our approach explored and quantified the multiplexity of the brain in two groups while performing an auditory oddball paradigm under the notion of a dynamic CFC approach. The features extracted for the training of the classifier were PAC values between frequency pairs at specific time windows that differed between the two groups (Figure 7). PAC values can be expressed as basic symbols of the neural syntax implying the efficiency or deficiency of coding of the cognitive content during a task-related stimulus (Buzsáki and Watson, 2012). PAC phenomenon can be interpreted as the formation of “packets” of higher frequency waves nested within the phase of the slower rhythms. At a quasi-stable time - window, the number of cycles of the higher frequency encapsulated within the phase of the slower frequency and this number is related to the amount of information being exchanged between different brain areas oscillating on their prominent frequency. According to the above interpretations of results, our approach bears some similarities with symbolic dynamics (Dimitriadis et al., 2012a, 2015b; Porta et al., 2015).

The frequency pairs that showed significant higher PAC value for MCI compared to NI group are the δ−α_2_, θ−α_2_, θ−β_1_, θ−γ, β_1_−γ and β_2_−γ (Figure 7). Previous studies demonstrated a decreased of δ amplitude in auditory tasks for MCI compared to the control group (Yener et al., 2012; Başar et al., 2013; Yener and Basar, 2013; Kurt et al., 2014). In this context, the higher coupling of δ phase with θ amplitude in MCI subjects can be interpreted as an increased attention (Dimitriadis et al., 2010a; Başar et al., 2013; Kurt et al., 2014). θ oscillations change during attention focusing (Sauseng et al., 2008), while the phase coupling in θ oscillation is known to reflect cognitive processes related to memory (Schack et al., 2002). In MCI participants, memory information which in general is stored within a distributed θ network, it is coupled with stronger PAC value compared to NI group with the amplitude of α_2_, β_1_, and γ frequencies showing the higher demands for MCI subjects to synchronize memory and attention state (Sauseng et al., 2008; Güntekin et al., 2013). In a recent study, based on recordings from rats Belluscio et al. (2012), showed that simultaneous maintenance of multiple items in working memory is accompanied by θ:γ phase-amplitude CFC in the hippocampus (Belluscio et al., 2012). Finally, phase of β sub-bands demonstrated a higher PAC synchronization with γ for MCI compared to NI group demonstrating high demands to shift the system to an attention state as a result of higher working memory load related to the counting mentally of the frequent tone. Overall, frequency-pairs that showed higher PAC values for MCI compared to the age-matched healthy group can be considered as a higher effort needed for MCI patients in order to perform accurately the auditory oddball task and due to overloaded cognitive systems related to attention and working memory. This hyper cross-synchronization observed in aMCI group is a significant finding of the current study. A previous MEG study where control and MCI group performed a memory task higher synchronization values were revealed over the parieto-occipital region in α and β frequency bands (Bajo et al., 2012a). Finally, the combination of memory tasks with connectivity analysis can differentiate healthy elderly from those with subjective memory complaints (Bajo et al., 2012b).

The main strengths of the present study are the significant MCI prediction improvement based on the proposed DCB, compared to standard techniques, and the single-sensor analysis methodology. Limitations of the study are the middle-sized sample of participants and adoption of an internal cross-validation scheme. Future studies will address those issues targeting a larger sample of MCI subjects, employing a second one for blinded classification and external cross-validation. Finally, a follow-up study for the subjects that progress to AD in the next 2 years will be of higher interest in order to explore the validity and the sensitivity of the proposed DCB to unfold the functional alterations, the inefficient organization of competing brain networks and the final integration breakdown due to the progression of the AD.

In summary, this study proves that the PAC in cognitive responses may be listed among the known functional changes due to MCI. Its quantification, maybe in conjunction with other CFC modes as well, can lead to reliable biomarkers. It is definitely worth further investigation, based on extended clinical cohorts and longitudinal data, so as to empirically prove that the PAC can serve as the basis of diagnostic and prognostic tools.

## Author contributions

Conception of the research: SD, NL, MB. Neuropsychological assessment: Maria Emiliana de Andrés. MCI evaluation: IT, MT. EEG data acquisition and database organization: IT. Methods design and data analysis: SD, NL, MB. Drafting of the manuscript: SD, NL, MB. Critical revision of the manuscript: IT, MT. Every author read and approved the final version of the manuscript.

### Conflict of interest statement

The authors declare that the research was conducted in the absence of any commercial or financial relationships that could be construed as a potential conflict of interest.
