# Supplementary material for: A novel biomarker of amnestic MCI based on dynamic cross-frequency coupling patterns during cognitive brain responses
Source: Front Neurosci. 2015 Oct 20;9:350. doi: 10.3389/fnins.2015.00350 (PMC4611062; doi:10.3389/fnins.2015.00350)

# A Short Guide to MATLAB code asscosiated with

# ''A novel biomarker of amnestic MCI

# based on dynamic Cross-Frequency Coupling patterns

# during cognitive brain responses''

***Frontiers in Neuroscience*** ,VOL.9, 2015, NUMBER=00350 [link](http://journal.frontiersin.org/article/10.3389/fnins.2015.00350/abstract) **DOI=10.3389/fnins.2015.00350**

edited by ***S.Dimitriadis & N.Laskaris***

**Neuroinformatics.Group, AUTH, Greece** [**site**](http://neuroinformatics.gr/)

Serving the spirit of reproducible research, we are including the main functions (m-files) and scripts developed for the above mentioned publication, along with a sample single-trial dataset, all the ensemble averages and all the derived PAC features based on which we applied our (machine-learning related) methdology for introducing the new biomarker.

There are three script-files , namely memo1,2&3, which demonstrate the main steps employed in our analysis. -The first one demonstrates the use of matlab ***rankfeatures*** command for estimating the discriminatory power of temporal patterning from the ensemble-average waveforms (provided as a mat-dataset).

- The second one exemplifies the use of PAC-estimator (described via eq.3 in our paper). The ***across-trial estimator*** is applied to single-trial responses from a normal subject (separately from ''target'' and ''non-target'' condition ) and the corresponding temporal profiles are compared (reproducing Fig.4 from our paper) .

- The third one exploits the PAC-traces that were computed for all subjects (and made availabe in FV_from_TV_PAC_analysis.mat) so as to select the more ''useful'' features for building an SVM-classifier. ***Feature selection*** and ***classifier*** application (training + testing) appear as distinct parts. In addition the code for reproducing Fig.7 is also provided.

Our code has been based on previous publications on the topic, which are aknowledged within the core m-file.

-Hoping it would become useful to similar neuroinformatic-explorations.


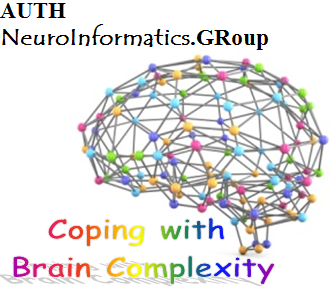

Supplement: Supplementary file 1 [file CFC_patterns_MCI_biomarker.ZIP › CFC_patterns_MCI_biomarker/Guide2_MatlabCode_4_CFC_based_MCI_detection.docx]
