# Supplementary material for: A novel biomarker of amnestic MCI based on dynamic cross-frequency coupling patterns during cognitive brain responses
Source: Front Neurosci. 2015 Oct 20;9:350. doi: 10.3389/fnins.2015.00350 (PMC4611062; doi:10.3389/fnins.2015.00350)
Supplement: Supplementary file 2 [file DataSheet1.DOCX]

# SUPPLEMENTARY MATERIAL

# A novel biomarker of amnestic MCI

# based on dynamic Cross-Frequency Coupling patterns

# during cognitive brain responses

S.I.Dimitriadis^1,2^, N.A.Laskaris^1,2^, Matina Bitzidou^1^, I. Tarnanas^3*^, M.N. Tsolaki^4^

^1^ AIIA lab, Informatics dept., AUTH, Greece

^2^ Neuroinformatics.Group, Informatics dept., AUTH, Greece

^3^ Health-IS Lab, Chair of Information Management, ETH Zurich, Zurich, Switzerland

^4^ 3^rd^ Department of Neurology, Medical School, AUTH, Greece

**S.1 Short-time-Fourier transform representation .**


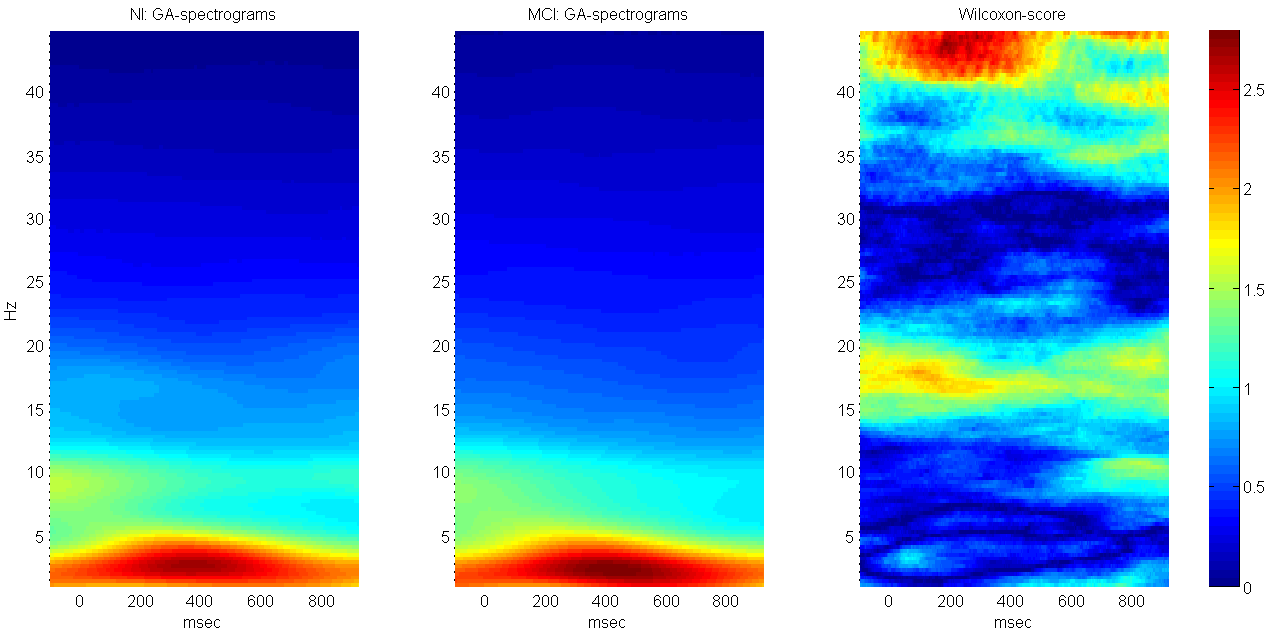


**Fig.S1** The left/middle panel portrays the Grand-averaged spectrograms from the cognitive responses of NI/aMCI participants. The shown maps correspond to power-spectral density estimated by means of the Welch’s method and have been brought to a common scale. The rightmost panel visualizes the separability score associated with each time-frequency point.

**S.2 Wavelet transform representation .**


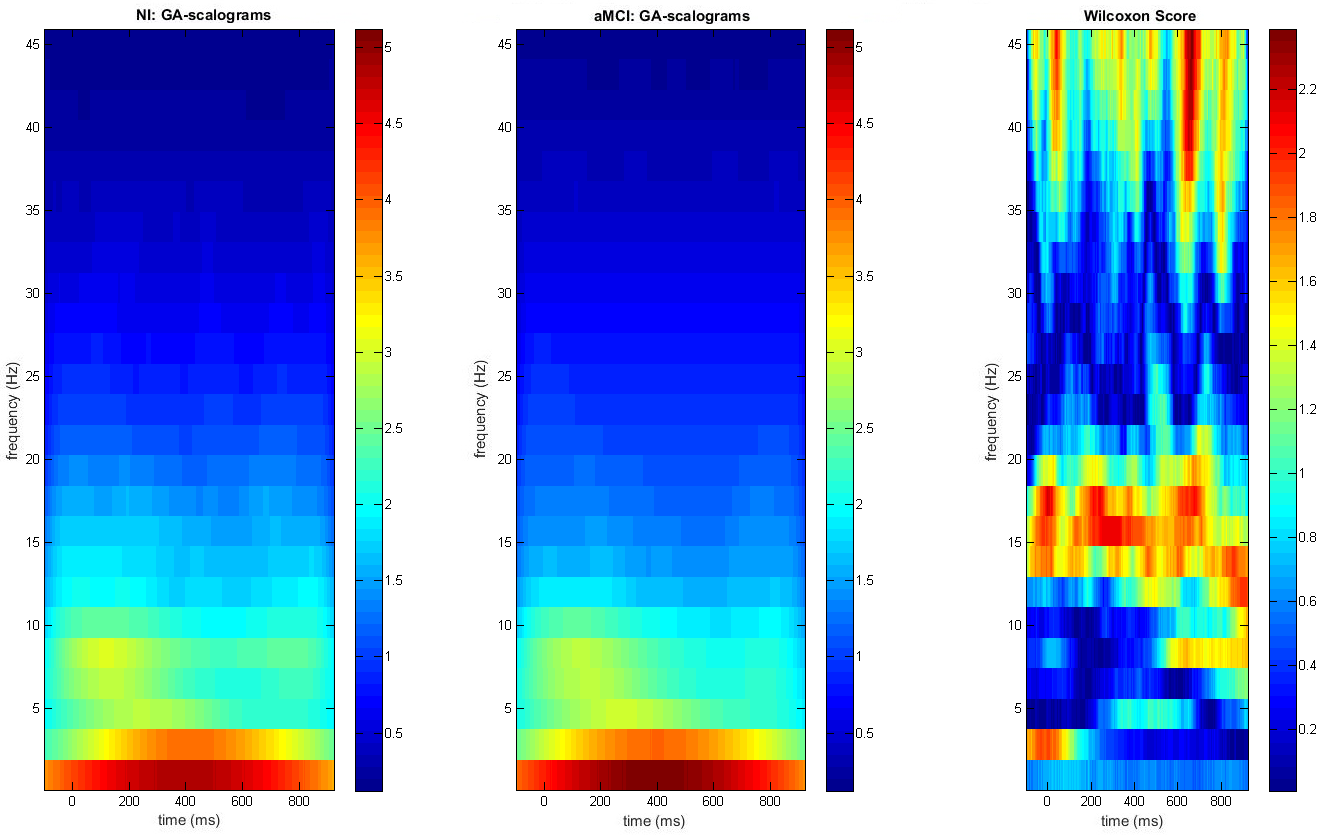


**Fig.S2** The Left/middle panel portrays the Grand-averaged scalogram from the cognitive responses of NI/aMCI participants. The shown maps correspond to the modulus from Morlet wavelet analysis and have been brought to a common scale. The rightmost panel visualizes the separability score associated with each time-frequency point.

The whole analysis was performed in Matlab via custom-made software.

The Matlab code with the meta-data (dynamic cross-frequency coupling estimates) will be uploaded in our websites (Dimitriadis’s homepage - <http://users.auth.gr/~stdimitr/index.html> ; Neuroinformatics Group - <http://neuroinformatics.gr/>) and also as a supplementary material in journal’s website.
